# Understanding user requirements to improve adoption of influenza diagnostics in clinical care within Metro Manila

**DOI:** 10.1002/hsr2.75

**Published:** 2018-08-07

**Authors:** Emily Gerth‐Guyette, Carol C. Malacad, Ma Paz Demonteverde, Dunia Faulx, Michael J. Lochhead, Socorro P. Lupisan, Brandon T. Leader, Veronica L. Tallo

**Affiliations:** ^1^ PATH Seattle WA USA; ^2^ Research Institute for Tropical Medicine Department of Health Metro Manila Philippines; ^3^ MBio Diagnostics, Inc. Boulder CO USA

**Keywords:** diagnostics, influenza, Manila, Philippines, products, users

## Abstract

**Background and aim:**

Influenza diagnostics play a critical role informing in clinical management decisions and defining the global epidemiology of the disease to support public health responses. Use of influenza diagnostics within most low‐income and middle‐income countries remains limited, including in the Philippines, where they are currently used only for epidemiologic surveillance. The aim of this study was to define key considerations, including product characteristics, which may influence future adoption, uptake, and integration of influenza diagnostics into public and private clinical settings in this emerging Asian market.

**Methods:**

Our study was conducted using a convenience sample of public and private hospital laboratories in Metro Manila. A usability assessment was conducted that included interviews with decision‐makers and direct observation of laboratory end users using 2 platforms representative of emerging diagnostic products: (1) a point‐of‐care antigen‐based rapid immunoassay diagnostic test paired with a reader and (2) a molecular diagnostic platform intended for decentralized use. Data were analyzed to assess user errors and device failure modes with each platform and to determine key considerations related to product adoption and uptake.

**Results:**

The most difficult test step for most users on both platforms involved sample preparation. When deciding to adopt a new test, priority product attributes include performance, potential volume of demand from clinicians, equipment cost, and ease of use. Demand for new tests is likely going to be driven by clinicians, and policies and guidelines will be needed to support the introduction of new products.

**Conclusion:**

Adoption of influenza diagnostics in Metro Manila is feasible but will require affordable products capable of satisfying needs for use in both epidemiologic surveillance and clinical management.

## 1 INTRODUCTION

Globally, viral respiratory infections—including influenza—are a leading cause of morbidity and mortality. Early and accurate diagnosis of influenza can inform better treatment decisions, including reducing the inappropriate use of antibiotics.^1^, ^2^ Influenza diagnostics are not commonly used in the majority of low‐income and middle‐income countries (LMICs), and clinicians rely on signs as well as on a consideration of seasonal epidemics and locally circulating viruses, to inform case management and treatment decisions. However, as influenza may be difficult to differentiate from other acute febrile illnesses, the collection of an appropriate sample and an accurate laboratory diagnostic test is required to establish a definitive diagnosis.^3^

While multiple options for influenza diagnostics exist, these tools vary considerably in accuracy, complexity, turnaround time, and other important performance characteristics.^4^ In addition, the necessary performance requirements of the diagnostic will depend on the context of use, be it for public health surveillance or to inform patient care at a referral hospital.^5^, ^6^ Recent evidence suggests that inappropriate or insensitive diagnostic assays may lead to the mismanagement of influenza cases.^7^ Moreover, reliance on central laboratories poses a challenge not only to improving patient case management but also to controlling nascent outbreaks and generating influenza surveillance data, particularly in places with limited laboratory capacity. This challenge is particularly evident in LMICs where laboratories have limited capacity and influenza epidemiology is poorly understood.^8^

The influenza A (H1N1) pandemic of 2009 exposed the limitations of available diagnostic tools to support large‐scale public health responses to influenza outbreaks.^9^ This diagnostic gap affected the capacity of the international community to quickly detect and respond effectively to this emerging infectious disease, particularly during the early stage of the pandemic. While the poor performance of rapid immunoassay diagnostic tests (RIDTs) for influenza during the 2009 H1N1 pandemic is well documented, there is compelling evidence regarding the improved performance of new tests, particularly molecular assays, and their potential to provide clinical utility.^10^, ^11^, ^12^, ^13^, ^14^, ^15^ While many of the new and emerging diagnostic tests for influenza offer superior performance to RIDTs, it not yet clear if these tests will adequately meet the needs of the end user. These needs may include supporting clinical case management, public health surveillance for seasonal influenza viruses, or emergency responses to pandemics.^16^ The cost, complexity, and turnaround times associated with these technologies often limit access outside of higher tier reference laboratories.^17^, ^18^

In both clinical case management and public health surveillance, as influenza diagnostics move closer to the patient and to the point of care (POC), the end user group also shifts, spurring a change in product design and development needs. It is critical to identify key product attributes that promote usability, as more complex diagnostics move into peripheral health laboratories and clinics. In addition to performance evaluations, usability assessments are needed to understand training requirements, whether target end users can successfully use the test and whether new tests can be feasibly integrated into health system policies and practices. This requires an assessment of next‐generation diagnostic tools in target use cases.

The Philippines represents an ideal setting for evaluation, given the potential need for new influenza diagnostic products within its growing health care market that includes both private and public segments. Also, it is located in an area of global importance for influenza. The Research Institute for Tropical Medicine (RITM) in the Philippines established an influenza surveillance program which includes sentinel sites in all regions across the country. Laboratory testing to support surveillance is conducted using respiratory specimens collected from patients identified at the peripheral health centers and outpatient departments of tertiary hospitals, that are then sent to RITM for testing via viral isolation techniques, and real‐time reverse transcriptase polymerase chain reaction using the US Centers for Disease Control and Prevention protocol.^19^ However, outside of RITM, routine influenza testing remains extremely limited in the Philippines. This is because, in part, of the lack of available diagnostic options with appropriate performance, cost, and user characteristics needed to expand the use of diagnostics for clinical care.

This study aimed to define key considerations, including product characteristics, which may influence future adoption, uptake, and integration of influenza diagnostics into public and private clinical settings in this emerging Asian market. Specifically, the study evaluated the usability and feasibility of introducing 2 influenza diagnostic platforms representative of emerging products—a POC RIDT paired with a reader and a moderately complex molecular diagnostic platform intended for decentralized use—among laboratory technicians in private and public health facilities.

## 2 METHODS AND MATERIALS

### 2.1 Study sites

**Figure 1:**
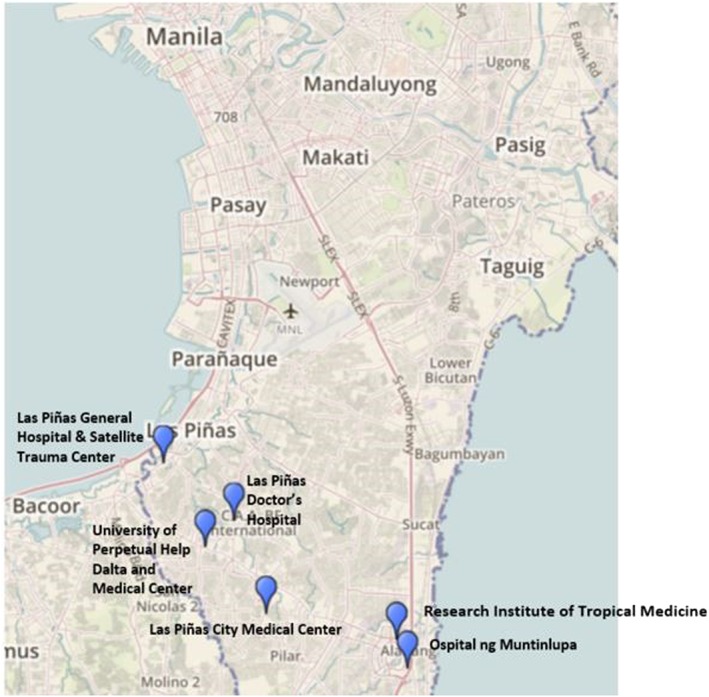
Map of study sites in Meto Manila

This study included a convenience sample of 6 hospitals in the Las Piñas and Muntinlupa areas of Metro Manila: Las Piñas City Medical Center, Las Piñas Doctor's Hospital, Las Piñas General Hospital & Satellite Center, Ospital ng Mutinlupa, Research Institute of Tropical Medicine, and University of Perpetual Help Dalta and Medical Center (Figure 1). The study team aimed to include health facilities in both the public and private sectors that were representative of various levels of laboratory capacity and that would likely be involved in screening and treating patients in the event of an influenza outbreak. Research Institute for Tropical Medicine was included, as it serves as the primary national reference facility for influenza testing for both clinical and surveillance purposes. Additional public and private hospitals were included to assess both surveillance and clinical case management use cases. Other eligibility criteria included willingness of hospital management to participate and whether the laboratory conducted rapid tests and employed a minimum of 10 technicians. Notably, many hospitals in the Metro Manila area meet these criteria, and the hospitals included here were, then, selected based on proximity to RITM (Table 1).

**Table 1:** Participating medical technicians randomized to 3 evaluation arms

Participating Hospitals	Total Participants Completed Run 1 and Run 2^a^	Molecular Platform Paper IFU	Molecular Platform Digital IFU	RIDT Paired with a Reader
1. RITM	22	6	8	8
2. Las Piñas City Medical Center	7	1	4	2
3. Las Piñas Doctors Hospital	6	3	2	1
4. Las Piñas General Hospital and Satellite Trauma Center	5	3	0	2
5. University of Perpetual Help DALTA Medical Center	4	1	1	2
6. Ospital ng Muntinlupa	2	1	1	0
Total	46	15	16	15

a Run 1 and run 2 refer to fact that each user included in the analysis ran the test twice. The initial run is referred to as run 1, and the second run (run 2) was conducted at the same facility, 24 hours later.

### 2.2 Participant characteristics

Six laboratory managers participated in this study. Laboratory technicians, rather than primary care providers, were the designated intended users for the tests. In the participating study hospitals, all diagnostic tests are conducted in the laboratory and at not at the bedside, regardless of complexity. Laboratory technicians often conduct rapid tests, such as dengue rapid tests. Forty‐six medical technicians (medtechs) participated in the evaluation of the influenza tests. Common responsibilities of medtechs include sample receipt, sample processing and testing, and specimen collection, including blood extraction, and releasing test results.

### 2.3 Study design

**Figure 2:**
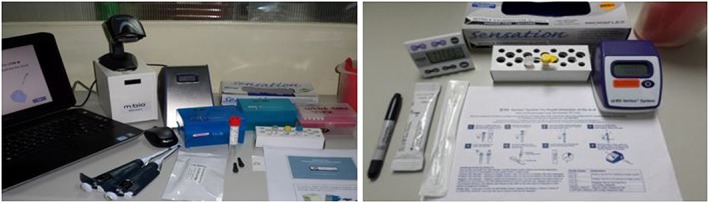
Influenza diagnostic platforms included in the study

This study used mixed methods to evaluate the usability and explore the feasibility of integrating near‐patient influenza diagnostic tests into clinical case management. To explore the feasibility of integrating near‐patient influenza diagnostic tests into clinical case management, key decision‐makers in the management of the laboratory at each hospital were recruited, to participate in an interview using a structured questionnaire. The questionnaire was piloted with the study team at RITM and allowed for “other” responses, to fully capture the participants' responses. The questionnaire focused on current testing procedures, laboratory systems and infrastructure, and how new tests are integrated into the laboratory. Convenience sampling was then used to recruit and enroll laboratory technicians to participate in the usability testing of the influenza diagnostics. Sampling was based on availability and willingness to participate and provide informed consent. To evaluate the usability of current influenza platform technologies, end users were observed performing the procedure of 1 of 2 representative near‐patient influenza diagnostic products intended for use at or near the POC (Figure 2):

1. A prototype molecular test: A prototype assay with a moderately complex molecular diagnostic platform intended for decentralized use, developed by MBio Diagnostics, Inc (Colorado, USA).
2. The BD Veritor: A commercially available POC RIDT (catalog number 256045) paired with a reader (catalog number 256055), developed by Becton Dickinson (BD) (New Jersey, USA).

Instructions for use (IFU) of the prototype molecular test were developed by PATH and MBio, vetted with laboratory technicians at RITM not involved in the study, and revised based on their feedback through an iterative process. Participants used either a digital or paper‐based version of the instructions. The paper‐based version of the instructions was printed and provided to users prior to running the test. The digital instructions were provided on a laptop computer on the bench; the content was identical to the paper‐based version, but only 1 step was shown at a time (arrows indicated where the user should click to move to the next step), and they included a built‐in timer for timed steps. The manufacturer's IFU included in the test kit was used for the BD Veritor. A randomization procedure was used to assign laboratory technicians to 1 of the 3 study arms: (1) the prototype molecular test using digital instructions, (2) the prototype molecular test using paper instructions, or (3) the BD Veritor RIDT.

Medical technicians performed the RIDT or the molecular test protocol and were asked to speak aloud what they were doing and thinking. No real samples were used; participants used water in place of specimen. The molecular test was programmed to give a contrived result, and the RIDT gave an invalid result. This testing procedure is referred to as the first run. They returned 24 hours later to run the same test protocol, referred to as the second run. User interactions were observed by the research team and captured with audio and video recording. Observations were recorded on standardized data collection forms by the research team and entered into an ACCESS database. These data were tabulated and counted to evaluate usability considerations and user errors at each step of the procedure.

Participants were interviewed about their experience using a semistructured exit interview guide focused on form factor, workflow processes, IFU, and overall impressions. Data from the interviews with laboratory managers and exit interviews with laboratory technicians were transcribed, double‐entered using ACCESS software, and coded based on themes related to usefulness, learnability, and user preferences. Data coded were reviewed by 2 members of the study team, categorized to develop ranked user preferences, and extracted to develop process maps.

The study protocol was reviewed and approved by the institutional ethical and scientific review committees at both PATH and RITM. Informed consent was obtained from all participants.

## 3 RESULTS

### 3.1 Feasibility of integrating near‐patient influenza diagnostic tests into clinical case management

While participants in the study had experience performing rapid diagnostic tests (RDTs) for infectious diseases including dengue, hepatitis, and HIV, familiarity with the use of rapid influenza diagnostics was limited across all sites. This included general familiarity with performing upstream procedural steps such as specimen collection. Sites commonly collected blood samples but rarely collected nasal or oropharyngeal swabs needed for influenza tests. In auxiliary equipment, users at all of the laboratories in the study had access to common clinical laboratory equipment while only the reference laboratory at RITM reported having polymerase chain reaction (PCR) machines. Because of the unscheduled demand for testing, all the laboratories were operational 24 hours a day, with shifts of technicians rotating every 8 to 12 hours.

**Figure 3:**
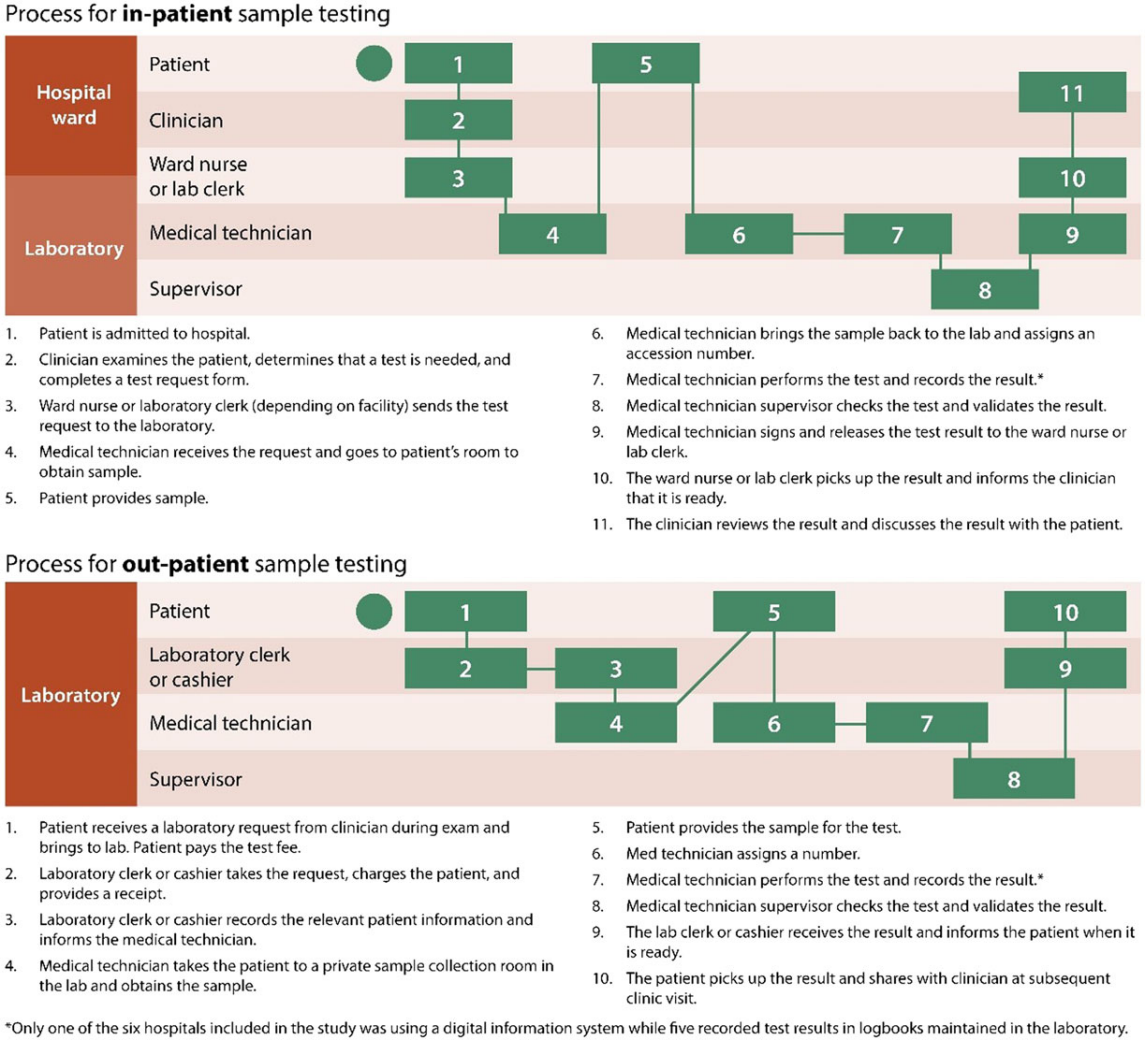
Process maps for inpatient and outpatient sample testing

To understand how near‐patient influenza diagnostics could be integrated into the clinic flow, laboratory managers were asked to describe the current processes and practices for laboratory testing at the facilities. Process maps (Figure 3) were generated based on their responses, which showed clear differences between inpatient and outpatient processes. Some differences were observed between private and public facilities. Notably, in outpatient private facilities, a cashier is involved to receive payment for the test. The cashier then uses the receipt of payment along with the physician's request to initiate the procedure, whereas in public or government facilities, no payment is required up front.

Currently, only the laboratory at RITM conducts confirmatory testing for influenza. Laboratory managers and the pathologist or head of the clinical laboratory are responsible for being aware of new technologies and also for making recommendations to the hospital administration regarding which tests should be added. They generally make a presentation and offer justification, including information on the cost of the investment as well as the equipment and training requirements. Table 2 outlines the reported factors that are taken into consideration when making procurement decisions.

**Table 2:** Factors considered most important by lab managers in the decision to procure diagnostic tests

Factors for Consideration	Government, n = 3	Private, n = 3	Total, n = 6
Sensitivity, specificity of the test	2	2	4
Potential volume of requests of the test by physicians	2	2	4
Cost of equipment	3	1	4
Cost of consumables	1	1	2
Ease in of performance	1	1	2
Turnaround time of the test	1	1	2
Whether the test is available in other hospitals	0	1	1
Availability of the equipment in the country	0	1	1

Overall, participants in both public and private facilities prioritized similar test characteristics when considering the implementation of new tests. Accuracy, defined as the sensitivity and specificity of the test, was an important consideration. Many of the participants were aware of the limitations of current rapid tests and emphasized that improved performance characteristics would be valued over the simplicity of rapid tests in their settings. Cost of equipment was a more prominent consideration for public facilities as all 3 public lab managers listed cost as the number 1 factor, compared to only 1 of the private lab managers (Table 2). Procurement decisions involved input from clinical staff, as lab managers indicated that their decisions would be driven by the testing services that doctors requested. “Ease of performance” was defined by managers as the estimated number of person‐hours required to perform the test procedure. This was a priority consideration expressed by 2 lab managers, who articulated that this would have to be accounted for when determining staffing and training needs. Laboratory managers also reported that the physicians were consulted before making a recommendation, and the head pathologist usually made final recommendations to the hospital administration and management regarding which diagnostic tests to procure.

There was general agreement that a near‐patient influenza diagnostic test would be useful to their work, and either test could be integrated into their current policies and practices. Laboratory managers noted that the value of the test would be as a screening test to rule out influenza during differential diagnoses and to ensure that only samples screened positive be subjected to confirmation with reference PCR testing for serotypes and lineages. Medtechs also reported some concerns regarding the general use of RIDTs, including test performance, test principle, and cost and maintenance requirements. Specific concerns regarding integration of tests into the laboratory include lack of familiarity with collection and handling of nasal swabs; biosafety, including the lack of biosafety hoods in some hospital laboratories; and the additional investments in staff needed for training and the performance of the test.

### 3.2 Usability of the representative influenza diagnostic platform technologies

Overall, all 46 medical technicians were able to perform the entire test procedure through to obtaining a result. The majority of user errors was self‐corrected and did not prevent users from completing the testing procedure. The most frequently observed user errors for both the molecular and RDT platforms occurred during sample preparation. Among the steps of the test procedure for both platforms, addition of external buffers to the test device was reported as the most difficult step. The RDT reader test took users about 7 minutes of “hands‐on” time for the first run while the molecular test took users about 15 minutes, again not including waiting times for incubations. In the second run, users on both platforms completed the test about 2 minutes faster. After running the tests, users outlined their preferences with regard to test form factor, workflow, and the accompanying IFU.

#### 3.2.1 Form factor

Users appreciated kit components that were easy to handle, light, compact, and portable. Users noted that while the RDT reader was compact enough to be used at the patient bedside, bedside testing is not currently conducted at any of the participating hospitals. Although the molecular platform required additional hardware components, users familiar with PCR machines noted that the required equipment was much smaller than other molecular platform products. Users appreciated that all ancillary supplies needed to run both tests were readily available in their laboratories and easy to reorder.

#### 3.2.2 Workflow

The molecular platform required a lengthier and more complex workflow compared with the RIDT reader. However, users reported that the workflow was acceptable and suggested that once a technician was familiar with the test, it could be done quickly and with a shorter turnaround time compared with conventional PCR. This is in line with managers' notion of ease of performance as defined primarily by the amount of time needed for use and training. Users of the RIDT reader suggested that after repeat use, they would likely no longer need to refer to the instructions. As all users ran only 1 sample, some questioned how well the platforms would accommodate batch testing. For both tests, a digital readout of the test result was preferred, as it provided a more definitive interpretation and increased confidence in the test result. A rapid turnaround time for results was considered a requirement for tests intended to inform patient care.

#### 3.2.3 Instructions for use

The availability of clear instructions with images and text was important, though preferences for digital or paper‐based instructions were mixed. Reported benefits of the digital IFU were the integrated timer and the fact that users did not need to move around paper instructions while handling test components. Drawbacks included a lack of familiarity with digital interfaces and the need to learn about a new test and a new IFU format at the same time. Users were clearly more familiar with paper instructions and required less intervention from the study team to help them move between steps. Users appreciated that both IFU relied on common words and short and direct sentences. Images reportedly helped users identify kit components and understand how they should be positioned.

## 4 DISCUSSION

While many publications have assessed performance characteristics of POC influenza tests, this paper looks outside of performance and provides some contextual detail related to test use and potential uptake in the context of clinical and surveillance settings in Metro Manila**.** At all of the laboratories, sufficient capacity was available both in skilled users and laboratory space to use both tests. Test users and laboratory supervisors were willing to consider the introduction of POC tests at their facilities. However, introducing either of the influenza diagnostic platforms was not replacing a current test but rather introducing a new diagnostic technology. As such, the value of the test needs to be demonstrated.

The results suggest that while hospital administrators and managers may execute procurement decisions, these decisions are driven by the use and the perceptions of tests among clinicians. In public health facilities, hospital administration and management responsible for procurement will rely on recommendations from pathologists and clinicians, who need to be assured of the reliability of the test results to inform clinical case management. Based on the process maps, as likely in other settings, multiple cadres of health workers needed to be familiar with new influenza tests, from the clinicians who recommend the tests to laboratory supervisors who need to validate the test results, and training needs may span across user groups involved in the continuum of testing.

The introduction of near‐patient influenza testing in high‐income settings has been found to be feasible and effective. Studies in emergency departments, pediatric departments, and other clinical settings have found that rapid influenza diagnosis has the potential to reduce costs associated with additional laboratory tests and radiographs, better target the use of antibiotics, antivirals, and infection control efforts, as well as shorten patient stays and prevent an overload of public health facilities during possible outbreaks.^1^, ^19^, ^20^ Similar demonstration studies are needed in LMICs to understand their full impact and cost implications. As new influenza diagnostics are introduced in this market, it will be important to ensure that all end users of both the tests and the test results have information on test performance, cost, and expected impact to generate demand and facilitate adoption.

While all participating medtechs had the requisite skills required to run both test platforms, some challenges were reported. Sample preparation steps posed the greatest difficulty for end users, followed by the addition of external buffers. The use of nasal swabs for sample collection and preparation was a reportedly new process for many of the participants. Introducing the use of a new sample type into laboratory procedures may require additional policy changes, including guidance on sample collection, transport, and storage. Current evidence suggests that trade‐offs exist between collection methods.^21^, ^22^, ^23^ Test developers looking to improve the usability of their tests may consider prioritizing innovations and features that simplify sample collection and preparation such as allowing for patient self‐swabbing, which has been demonstrated to be both effective and acceptable among patients.^24^, ^25^, ^26^

Overall, the value and potential of new platform technologies for influenza diagnosis were recognized both for clinical case management in health facilities and to improve influenza surveillance and monitoring at RITM. However, given indications from managers that even molecular POC tests would most likely be used as a screening test in the near term, reference laboratories such as RITM may still be required for a confirmatory diagnosis. A recent editorial regarding the need for better influenza testing suggests that even when clinical guidelines enable antiviral treatment based on improved POC tests, a gap exists between these guidelines and clinical treatment practices.^27^ Thus, the value of new tests in Manila and elsewhere may be limited until global and national guidelines are changed to reflect the role these tools can play in both screening and case management, and until clinicians adhere to these evolving guidelines.

This evaluation had several key limitations. For one, this usability assessment did not include the use of actual specimens or specimen collection, which may impact usability and feasibility of adoption. Additionally, these platforms may require new systems and processes to accommodate the use of a potentially infectious specimen collected at the POC or near the patient. Furthermore, the adoption and use of new diagnostic platforms is highly contextual, and the findings of this study may not be applicable to other contexts, particularly in peripheral settings where access to health services and well‐trained laboratorians are more limited. While the results of this study suggest that these tests could become part of routine practices with minimal training requirements, the medical technicians working in both settings were skilled, with several years of experience. This may or may not be the case in more peripheral health care settings with lower skilled medical technicians. This study sample is not exhaustive of all settings where better influenza diagnostics are needed.

## 5 CONCLUSION

Although influenza diagnostics were not currently in use by any of the participating hospitals apart from RITM, results of the study indicated that the potential for influenza tests to be used within other laboratories in the Philippines is high. Priority attributes of future influenza diagnostic products should satisfy criteria considered important by laboratory managers: improved sensitivity and specificity over current rapid tests, high volume of tests demanded by clinicians, and low cost of equipment.

The impact of any improved diagnostic will be determined by whether the test is used for screening, differential diagnosis, or to inform treatment decisions, which will, in turn, be mediated by both official guidelines and clinical practice. Public and private clinical settings in Metro Manila require products that are accurate and affordable and which ideally can be used to support both clinical management and surveillance use cases. Integrating near‐patient or POC influenza diagnostics into clinical care has the potential to not only improve data informing the global epidemiology of the disease but also strengthen clinical decision‐making, including informing more appropriate antibiotic use.

## ACKNOWLEDGEMENTS AND DISCLOSURES

PATH would like to thank the laboratory managers and medical technicians for their participation in this project. Research reported in this publication was supported by the National Institute of Allergy and Infectious Diseases of the National Institutes of Health under award number R01AI96189. The content is solely the responsibility of the authors and does not necessarily represent the official views of the National Institutes of Health. MJL is an employee of MBio Diagnostics, Inc. The authors declare no relevant financial interest related to this study.

## CONFLICT OF INTEREST

The authors have declared that there is no conflict of interest.

## AUTHOR CONTRIBUTIONS

Conceptualization: DF, MJL, SPL, BTL, VLT

Formal analysis: EGG, CCM, MPD, VLT

Funding acquisition: MJL, SPL, BTL, VLT

Investigation: EGG, CCM, MPD, VLT

Methodology: EGG, DF, MJL, BTL, VLT

Project administration: CCM, MPD, VLT

Resources: MJL

Software: MJL

Supervision: DF, VLT

Writing—original draft preparation: EGG, BTL, VLT

Writing—review and editing: EGG, BTL, VLT
